# An 81 base-pair deletion in SARS-CoV-2 ORF7a identified from sentinel surveillance in Arizona (Jan-Mar 2020)

**DOI:** 10.1101/2020.04.17.20069641

**Published:** 2020-04-22

**Authors:** LaRinda A. Holland, Emily A. Kaelin, Rabia Maqsood, Bereket Estifanos, Lily I. Wu, Arvind Varsani, Rolf U. Halden, Brenda G. Hogue, Matthew Scotch, Efrem S. Lim

**Affiliations:** 1 Center for Fundamental and Applied Microbiomics, Biodesign Institute, Arizona State University, Tempe, Arizona, USA.; 2 School of Life Sciences, Arizona State University, Tempe, Arizona, USA.; 3 Center for Immunotherapy, Vaccines and Virotherapy, Biodesign Institute, Arizona State University, Tempe, Arizona, USA.; 4 Center of Evolution and Medicine, Arizona State University, Tempe, Arizona, USA.; 5 Structural Biology Research Unit, Department of Integrative Biomedical Sciences, University of Cape Town, Observatory, Cape Town 7701, South Africa; 6 Center for Environmental Health Engineering, Biodesign Institute, Arizona State University, Tempe, Arizona, USA.; 7 One Water One Health Nonprofit Project, Arizona State University Foundation, Tempe, Arizona, USA.; 8 Center for Applied Structural Discovery, Biodesign Institute, Arizona State University, Tempe, Arizona, USA.; 9 College of Health Solutions, Arizona State University, Phoenix, Arizona, USA.

On January 26 2020, the first Coronavirus Disease 2019 (COVID-19) case was reported in Arizona of an individual with travel history (3^rd^ case in the US) (1). Here, we report on early SARS-CoV-2 sentinel surveillance in Tempe, Arizona (USA). Genomic characterization identified an isolate encoding a 27 amino acid in-frame deletion in accessory protein ORF7a, the ortholog of SARS-CoV immune antagonist ORF7a/X4.

In anticipation of COVID-19 spreading in the state of Arizona, we initiated a surveillance effort for local emergence of SARS-CoV-2 starting January 24, 2020. We leveraged an ongoing influenza surveillance project at Arizona State University (ASU) Health Services in Tempe, Arizona. Individuals presenting with respiratory symptoms (ILI) were tested for influenza A and B virus (Alere BinaxNOW). Subsequently, we tested influenza-negative nasopharyngeal (NP) swabs for SARS-CoV-2. We extracted total nucleic acid using the bioMérieux eMAG automated platform and performed real-time RT-PCR (qRT-PCR) assays specific for SARS-CoV-2 N and E genes (2, 3). Out of 382 NP swabs collected from January 24, 2020 to March 25, 2020, we detected SARS-CoV-2 in 5 swabs in the week of March 16 to 19 (Figure 1). This corresponds to prevalence of 1.31%. Given the estimated 1 – 14-day incubation period for COVID-19, it is possible that the spike in cases might be related to university spring-break holiday travel (March 8 – 15) as previously seen in other outbreaks (4, 5).

To understand the evolutionary relationships and characterize the SARS-CoV-2 genomes, we performed next-generation sequencing (Illumina NextSeq, 2×76) directly on specimen RNA, thereby avoiding cell culture passage and potentially associated mutations. This generated an NGS dataset of 20.7 to 22.7 million paired-end reads per sample. We mapped quality-filtered reads to a reference SARS-CoV-2 genome (MN908947) using BBMap (version 39.64) to generate three full-length genomes: AZ-ASU2922 (376x coverage), AZ-ASU2923 (50x) and AZ-ASU2936 (879x) (Geneious prime version 2020.0.5). We aligned a total of 222 SARS-CoV-2 genome sequences comprising at least 5 representative sequences from phylogenetic lineages defined by Rambaut *et al.* (6), ranging from January 5 to March 31, 2020 from 25 different countries. We performed phylogenetic reconstruction with BEAST (version 1.10.4, strict molecular clock, HKY + Γ nucleotide substitution, exponential growth for coalescent model) (7–10). The ASU sequences were phylogenetically distinct indicating that they were independent transmissions (Figure 2A).

Similar to SARS-CoV, the SARS-CoV-2 genome encodes multiple open reading frames in the 3’ region. We found that the SARS-CoV-2 AZ-ASU2923 genome has an 81 base-pair deletion in the ORF7a gene resulting in a 27 amino-acid in-frame deletion (Figure 2B). The SARS-CoV ORF7a ortholog is a viral antagonist of host restriction factor BST-2/Tetherin and induces apoptosis (11–14). Based on the SARS-CoV ORF7a structure (15), the 27-aa deletion in SARSCoV-2 ORF7a maps to the putative signal peptide (partial) and first two beta strands. To validate the deletion, we performed RT-PCR using primers spanning the region and verified by Sanger sequencing the amplicons (Figure 2C and Supplementary Figure 1).

Collectively, although global NGS efforts indicate that SARS-CoV-2 genomes are relatively stable, dynamic mutations can be selected in symptomatic individuals.

## Supplementary Material

1

## Figures and Tables

**Figure 1:**
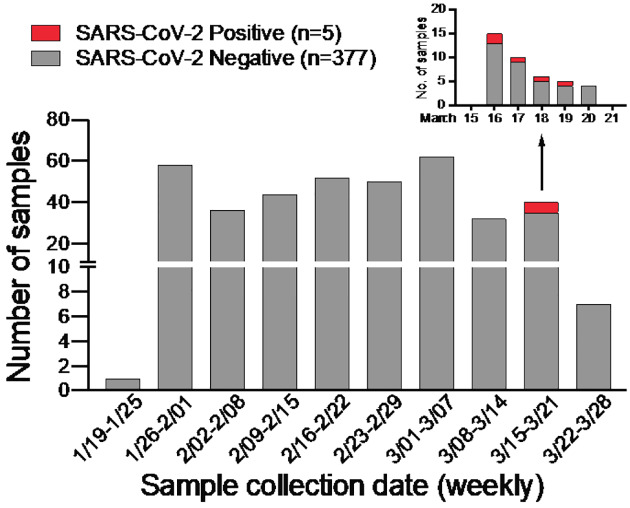
SARS-CoV-2 surveillance in Tempe, AZ from January to March 2020. Weekly distribution of NP specimens collected by ASU Health Services tested for SARS-CoV-2 by qRT-PCR assays. Inset shows SARS-CoV-2 positive NP specimens collected from the week of March 15 – 21, 2020.

**Figure 2:**
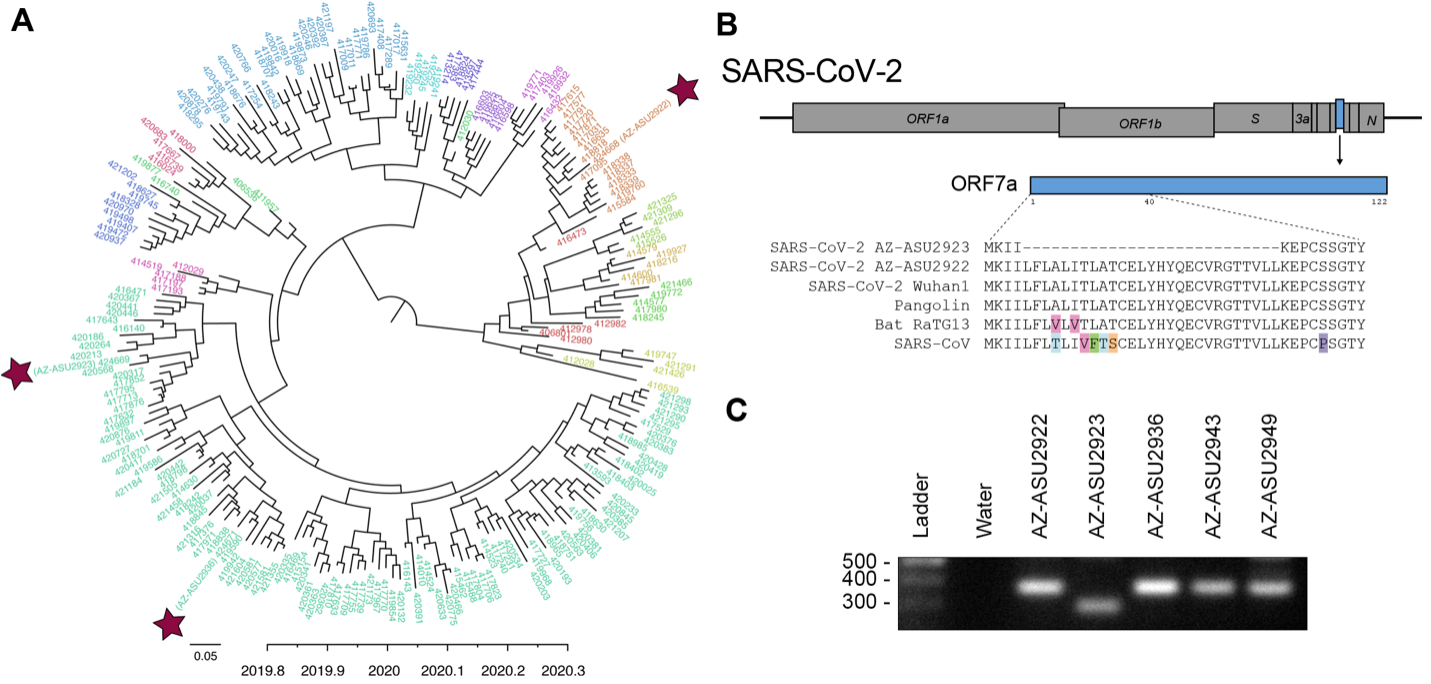
Evolutionary and genomic characterization of SARS-CoV-2 genomes. (**A**) Bayesian maximum clade credibility (MCC) polar phylogeny of 222 full-length SARS-CoV-2 genomes. The 3 new genomes reported in this study are indicated by red stars. Sequenced were aligned in Geneious prime (version 2020.0.3) using the MAFFT v7.450 plugin, and trimmed the 5’ and 3’ UTR (< 300 nts each). We initiated two independent runs of 500M sampling every 50K steps and used Tracer v1.7.1 (16) to check for convergence and that all ESS values for our statistics were > 200, LogCombiner (7) to combine the models with a 10% burn-in and TreeAnnotator (7) to produce a MCC tree. We used FigTree v1.4.4 (17) to edit the tree and color the tips based on lineages (6), and pangolin (18) to identify the lineages of our 3 new sequences based on the established nomenclature (6). The nomenclature consists of two main lineages, A and B, and includes “sub-lineages” (A.1, B.2. *etc*.) up to four levels deep (e.g. A.1.1, B.2.1) (6). For visualization purposes, we grouped all viruses that were not directly assigned to “A” or “B” into their first sub-lineage level and colored tip labels by lineage. B.1 lineage: AZ-ASU2923 and AZ-ASU2936; A.1 lineage AZ-ASU2922. (**B**) ORF7a amino acid alignment of SARS-CoV-2 and related genomes. GenBank and GISAID accession numbers: SARS-CoV-2 AZ-ASU2922 (MT339039, EPI_ISL_424668), SARS-CoV-2 AZ-ASU2923 (MT339040, EPI_ISL_424669), SARS-CoV-2 AZ-ASU2936 (MT339041, EPI_ISL_424671), SARS-CoV-2 Wuhan1 (MN908947.3), Pangolin (EPI_ISL_410721), Bat-RaTG13 (MN996532.1), SARS-CoV (AY278741.1). The 81-bp (27 amino acid) deletion observed in SARS-CoV-2 AZ_ASU2923 ORF7a was not present in the 6,290 SARS-CoV-2 sequences available from GISAID as of April 12, 2020. (**C**) We performed molecular validation by RT-PCR on specimen total nucleic acid extracts with primers flanking the ORF7a N-terminus region. The expected size of amplicons with intact ORF7a region is 377bp, the expected size of an amplicon with the NGS-identified 81bp deletion is 296bp. Primers: SARS2–27144F 5’-ACAGACCATTCCAGTAGCAGTG-3’, SARS2–27520r 5’-TGCCCTCGTATGTTCCAGAAG-3’.
