# Supplementary material for: An 81 base-pair deletion in SARS-CoV-2 ORF7a identified from sentinel surveillance in Arizona (Jan-Mar 2020)
Source: medRxiv. 2020 Apr 22:2020.04.17.20069641. Preprint. [Version 1] doi: 10.1101/2020.04.17.20069641 (PMC7276018; doi:10.1101/2020.04.17.20069641)
Supplement: 1 [file NIHPP2020.04.17.20069641-supplement-1.pdf]

medRxiv preprint doi: https://doi.org/10.1101/2020.04.17.20069641.this version posted April 22, 2020. The copyright holder for this preprint (which was not certified by peer review) is the author/funder, who has granted medRxiv a license to display the preprint in perpetuity.
It is made available under a CC-BY 4.0 International license .

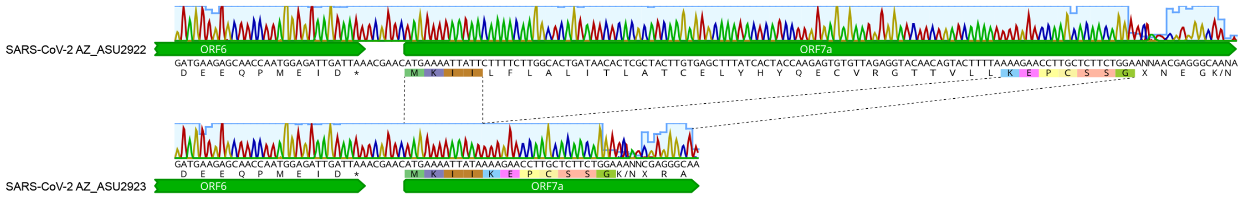

Supplementary Figure 1. Sanger sequencing chromatograms verification of 81-bp deletion in SARS-CoV-2 AZ-ASU2923 in the ORF7a region.
